# 

**Published:** 1841-07

**Authors:** 


### QUARTERLY JOURNAL

OF

### PRACTICAL MEDICINE AND SURGERY

LIBRARY OF THE

Orleans Parish Medical Society

EDITED BY

JOHN FORBES M.D. F.R.S. F.G.S.

VOL. XII.

JULY—OCTOBER 1841

LONDON
JOHN CHURCHILL PRINCES STREET SOHO.
MDCCCXLI.

LONDON:

PRINTED BY C. ADLARD, BARTHOLOMEW CLOSE.

### BOOKS RECEIVED FOR REVIEW.

BRITISH.

1. Memoir on the Radical Cure of Stuttering by a Surgical Operation. By J. F. Dieffenbach. Translated from the German by Joseph Travers, late House-surgeon to St. Bartholomew's Hospital.—London, 1841. 8vo, pp. 26. Plates. 3s.

2. Guy's Hospital Reports. No. XII., April, 1841. 6s.

3. Elements of Medicine. Vol. II. On Morbid Poisons. By Robert Williams, M.D., Senior Physician to St. Thomas's Hospital.—Lond. 1841. 8vo, pp. 686. 18s.

4. The Anatomy of the Arteries of the Human Body. By Richard Quain and Joseph Maclise. Part VI. 8vo and folio. April, 1840. 12s.

5. The Baths of Central and Southern Germany, considered with reference to their remedial efficacy in Chronic Disease. By E. Lee, Esq.—London and Paris, 1841. 8vo, pp. 139. 3s. 6d.

6. A Complete Practical Treatise on Venereal Diseases, and their immediate and remote Consequences; including observations on certain Affections of the Uterus attended with Discharges. By William Acton.—London, 1841. 8vo, pp. 410. With an Atlas of Plates. 1*l.* 11s. 6d.

7. A Medical Guide to Nice; containing every information necessary to the Invalid and Resident Stranger. By W. Farr, M.D. —London, 1841. 8vo, pp. 177. 5s. 6d.

8. On the Cure of Strabismus. By Thomas Elliott. (From the Edinburgh Journal.) 8vo, pp. 27.

9. Jaundice from Non-Elimination. By W. Lowe, M.D. (From the Edinburgh Journal.) 8vo, pp. 19.

10. Report of the Lunatic Department of the Edinburgh Charity Workhouse for 1836-7-8. By John Smith, M.D. 8vo, pp. 12.

11. Report of the Saughton Hall Lunatic Asylum. Nov. 1840. 8vo, pp. 12.

12. Stammering and other Imperfections of Speech treated by Surgical Operations on the Throat, &c. By J. Yearsley, M.R.C.S.—London, 1841. 8vo, pp. 46. 1s.

13. The Philosophy of Mystery. By W. C. Dendy, F.L.S.—London, 1841. 8vo, pp. 443. 12s.

14. The Madras Quarterly Medical Journal. Edited by Samuel Rogers. Vol. II.—Madras, 1840. 8vo, pp. 462.

15. Hints for Invalids about to visit Naples; being a sketch of the Medical Topography of that city. Also, an account of the Mineral Waters of the Bay of Naples. By J. C. Cox, M.D., F.L.S.—London, 1841. 8vo, pp. 190. 7s. 6d.

16. Popular Cyclopædia of Natural Science. Vegetable Physiology.—London, 1841. 8vo, pp. 295.

17. Researches on Operative Midwifery, &c. By Fleetwood Churchill, M.D.—Dublin, 1841. 8vo, pp. 325. With plates. 14s.

18. On the Minute Structure and Movements of Voluntary Muscle. By William Bowman, Esq. (From the Philosophical Transactions.)—London, 1840.

19. The Transactions of the Provincial Medical and Surgical Association. Vol. IX.—London, 1841. 8vo, pp. 583. 21s.

20. Spinal Affections: A Popular Lecture on Disorders and Diseases of the Spine. By H. C. Roods, M.C.S.L.—London, 1841. 12mo, pp. 57. 2s.

21. The Philosophy of Death; or a General Medical and Statistical Treatise on the Nature and Causes of Human Mortality. By John Reid, Licentiate of the Faculty of Physicians and Surgeons of Glasgow.—London, 1841. 8vo, pp. 381. 6s. 6d.

22. Report of the Cases attended at the Birmingham Eye Infirmary during the years 1838-9. By R. Middlemore, Esq. (From the Transactions of the Provincial Association.)—Worcester, 1841. 8vo, pp. 20.

23. Rambles in Europe in 1839; with sketches of prominent Surgeons, Physicians, Medical Schools, &c. By W. Gibson, M.D., Professor of Surgery in the University of Pennsylvania, &c.—Philadelphia, 1841. 8vo, pp. 309.

24. The Sanative Influence of Climate; with an account of the best Places of Resort for Invalids, in England, the South of Europe, &c. By Sir James Clark, Bart., M.D., F.R.S., Physician to the Queen. 3d edit.—Lond., 1841. 8vo, pp. 377. 10s. 6d.

25. Eleventh Annual Report of the Belfast District Asylum for Lunatic Poor.—Belfast, 1841. 8vo, pp. 26.

26. A General Outline of the Animal Kingdom, and Manual of Comparative Anatomy. By T. Rymer Jones, Professor of Comparative Anatomy in King's College. With 336 engravings.—London, 1841. 8vo, pp. 732. 1*l.* 18s.

27. Report upon the Mortality of Lunatics. By W. Farr, Esq. (From the Journal of the Statistical Society.)—London, 1841. 8vo, pp. 17.

28. A Treatise on Pyrosis Idiopathica, or Water Brash, as contrasted with certain forms of Indigestion, &c. By Thomas West, M.D.—Lond. 1841. 8vo, pp. 108. 5s.

29. Observations on the Surgical Pathology and Treatment of Aneurism; being the substance of a course of lectures on that disease. Part I. By W. H. Porter, A.M., Surgeon to the Meath Hospital, Dublin. (No date.) 8vo, pp. 214. 6s.

30. On certain Physiological Inferences which may be drawn from the study of the Nerves of the Eyeball. By W. P. Alison, M.D., F.R.S.E. (From the Transactions of the Royal Society of Edinburgh.)—Edinburgh, 1841. 4to, pp. 20.

31. An Essay on the Chemical, Botanical, Physical, and Parturient Properties of the Secale Cornutum. By T. H. Wardleworth, surgeon.—London, 1840. 12mo, pp. 69.

32. The Physiology of Vision. By William Mackenzie, M.D., Lecturer on the Eye in the University of Glasgow.—London, 1841. 8vo, pp. 292. 10s. 6d.

33. Odontography; or, a Treatise on the Comparative Anatomy of the Teeth. By Richard Owen, F.R.S. Part II., with 50 plates.—London, 1841. Royal 8vo. 31s. 6d.

34. On Stammering and Squinting, and on the Methods for their Removal. By Edwin Lee, M.R.C.S., &c.—London, 1841. 8vo, pp. 88. 3s.

35. Phrenology consistent with Science and Revelation. By Charles Cowan, M.D., &c. 12mo, pp. 55.—Reading and London, 1841. 2s. 6d.

36. An Inquiry concerning the Diseases of the Brain, the Spinal Cord, and the Nerves. By Amariah Brigham, M.D.—New York, 1840. 8vo, pp. 327.

